# A new model of time scheme for progression of colorectal cancer

**DOI:** 10.1186/1752-0509-8-S3-S2

**Published:** 2014-10-22

**Authors:** Shuhao Sun, Fima Klebaner, Tianhai Tian

**Affiliations:** 1 School of Mathematical Sciences, Monash University, VIC 3800 Melbourne, Australia

## Abstract

**Background:**

tumourigenesis can be regarded as an evolutionary process, in which the transformation of a normal cell into a tumour cell involves a number of limiting genetic and epigenetic events. To study the progression process, time schemes have been proposed for studying the process of colorectal cancer based on extensive clinical investigations. Moreover, a number of mathematical models have been designed to describe this evolutionary process. These models assumed that the mutation rate of genes is constant during different stages. However, it has been pointed that the subsequent driver mutations appear faster than the previous ones and the cumulative time to have more driver mutations grows with the growing number of gene mutations. Thus it is still a challenge to calculate the time when the first mutation occurs and to determine the influence of tumour size on the mutation rate.

**Results:**

In this work we present a general framework to remedy the shortcoming of existing models. Rather than considering the information of gene mutations based on a population of patients, we for the first time determine the values of the selective advantage of cancer cells and initial mutation rate for individual patients. The averaged values of doubling time and selective advantage coefficient determined by our model are consistent with the predictions made by the published models. Our calculation showed that the values of biological parameters, such as the selective advantage coefficient, initial mutation rate and cell doubling time diversely depend on individuals. Our model has successfully predicted the values of several important parameters in cancer progression, such as the selective advantage coefficient, initial mutation rate and cell doubling time. In addition, experimental data validated our predicted initial mutation rate and cell doubling time.

**Conclusions:**

The introduced new parameter makes our proposed model more flexible to fix various types of information based on different patients in cancer progression.

## Background

Carcinogenesis is the transformation of normal cells into cancer cells. This process has been shown to be of a multistage nature. It involves somatic mutations in any cellular genome, either induced by external source or spontaneously occurring during the mitotic replication, and thus it may comprise different types of DNA alterations. Moreover, the cell genome may acquire entire sequences from exogenous sources. The epigenetic changes, which alter chromatin structure, can also be subject to the same selection forces as genetic events [1-4].

Colorectal tumourigenesis proceeds through a number of well defined clinical stages. The process is initiated when a single colorectal epithelial cell acquires a mutation in a gene that inactivates the APC/*β*-catenin pathway. Mutations that constitutively activate the K-Ras/B-Raf pathway are associated with the growth of a small adenoma to a clinically significant size (namely > 1 cm in diameter).

Subsequent waves of clonal expansion driven by mutations in genes controlling the TGF-*β *[5], PIK3CA [6], TP53 [7], and other pathways are responsible for the transition from a benign tumour to a malignant tumour. Some tumours eventually acquire the ability to migrate and seed other organs [1]. In general, it is still quite difficult to give a precise definition of the steps in the evolutionary process.

The mathematical investigation of cancer started in early 1950s [8,9], aiming at deriving the basic laws regarding the tumour dynamics and elaborating a comprehensive framework for testing hypothesis [10-14]. Later on, other types of experimental evidence, based on epidemiological data of cancer mortality, allowed the development of the multistage theory (MST) of cancer development [15-18]. Among them, population genetics models are used extensively to describe tumourigenesis [16,19-21], since cancer progression is an evolutionary process. Various deterministic and stochastic models have been proposed. Some models addressed specific questions, such as the dynamics of tumour suppressor genes [22-25], genetic instability [26,27], or tissue architecture [20]. computer Computer simulations [28] and theoretical analysis [29,30] have been employed to investigate the properties of these models In particular, the stochastic multistage cancer model is a well-known model of cancer development [3,18,31,32]. Models of tumourigenesis have been proposed early on to explain cancer incidence data [33-36]. In a series of studies, Berenblum and Shubik proposed the "two-stage" model of carcinogenesis [37]. These models assumed that cancer is a stochastic multistep process with small transition rates and they have been further developed into the multistage theory of cancer [17,38-40]. Multistage models provide a natural framework to evaluate the potential benefits of prevention and intervention strategies designed to reduce cancer risk. A comprehensive review on this topic can be found in [41].

Beerenwinkel et al. proposed one of the first mathematical models that explicitly used genome data to simulate the somatic evolution of colorectal tumour [15]. The proposed model investigated the importance of selection, as the driving force in tumour progression, from a benign tumour (~ 1 mg or 10^6 ^cells) to a full-grown cancer (~ 1 gram or 10^9 ^cells), over a period of 5-20 years. The tumourigenic progression is described as the Wright-Fischer process, where cells evolve in non-verlapping generations and each new cell can acquire a new mutation with probability "*u*" at each non-mutated genelocation. It has been derived that the time period of the *k*-th mutation is given by

(1) $$t_{k}=k\frac{{\left(\text{ln}\frac{s}{ud}\right)}^{2}}{2s\text{ ln }N}$$

where *s *is the advantageous selection coefficient, *u *the rate of driver mutations, *d *the number of sensitively mutated genes, *k *the number of susceptible genes (potential drivers), and *N *is the size of cell population. Based on the Galton-Watson branching process and investigation in [15], a mathematical model was developed to conciliate cancer genomic data with epidemiologic and clinic observations [42]. The model dynamics depends on only three parameters: namely the driver mutation rate (*u*), the selective advantage associated with driver mutation (*s*), and the cell division time (*T*). Using an estimated averaged mutation rate per cell division of 3.4 · 10^−5^, the averaged time for the acquisition of subsequent driver mutationsis is given by

(2) $$t_{k}=\frac{T}{ks}\text{ln}\frac{2ks}{u}$$

where T is the average time of the cell division.

Based on this formula, it was calculated that the actual selective advantage provided by typical somatic mutations in *human tumours in situ *was 0.004 ± 0.0004. However, formula (2) does not involve the tumour size and the waiting time was independent of the population size. Thus it is not appropriate to use it to calculate the time required for the first mutation appearance.

Extending these observations, by sing the approach of "comparative lesion sequencing", Jones et al. [1] examined quantitatively the mutations described in colon cancers genomic studies and in other neoplastic lesions of the same patients. They estimated the time intervals required for the appearance of the cells that originate the clonal expansions. In this model it was assumed that, in each carcinogenic stage (namely micro-adenoma, small and large adenoma, early and advanced carcinoma, and metastasis), there is a founder cell that gives rise to the various tumour populations. During tumour evolution, cells acquire other mutations and may become founder cells of succeeding carcinogenic states. For example, the time interval between the birthdate of a founder cell for a large adenoma and the founder cell of an advanced carcinoma can be approximated as.

(3) $$\text{Δ}T_{LAd,ACa}=F_{Lad,Aca}\cdot T_{ACa},$$

where *F_Lad,Aca _*is the fraction of mutations in the advanced carcinoma that were not found in the large adenoma and *T_ACa _*is the birthdate of the founder cell of advanced carcinoma.

In the same line, Yachida et al. used genomic data of seven pancreatic cancer patients presenting metastases in [43] to evaluate the clonal relationships among primary and metastatic cancers. The results showed that the pattern that originated metastasis were clearly represented in the cells within the primary carcinoma (i.e., itself) in the form of a combination of many distinct subclones. In addition, each subclones has its own heterogenic pattern. Using the following mathematical model:

$$T=\frac{T_{gen}}{r}\left(N_{1}+\sqrt{N_{1}}\right)$$

where *T_get _*= 2.3 days and *r *= 0.016 per generation, they calculated the elapsed time between the different stages of the tumourigenic process. The results showed an averaged time of 11.7 years from the initiation of tumourigenesis to the birth of the cell giving rise to the parental clone, an averaged time of 6.8 years from then to the birth of the cell giving rise to the index lesion, and an averaged time of 2.7 years from then until the patients' death. This result is consistent with that reported in [1]. Taking these correlations together, the dynamics of the tumour progression offers an opportunity to interfere in the tumour evolution and develop a more customized treatment.

However, the information about the initial mutation rate and selective advantage of tumour cell for each individual patient have not been revealed yet in all studies reviewed above. This information is very important for cancer treatment. Although we conducted studies to describe cancer progression for a number of individuals [44], the results were not satisfactory. Thus in this work we will develop a more general framework that not only maintains the advantage of existing mathematical models but also is able to calculate individual patient's initial mutation rate and selective advantage of tumour cell which has potential applications for medical treatment.

## Results and discussion

### A general modelling framework

According to the multistage theory, cancer is the last stage of a series of *k *sudden and irreversible changes which must take place in a cell in a specific order. Denote state *i *is that a cell has i mutations, *p_i_*(*t*) is the fraction of all cells in state *i *in the whole population, and *µ*(*t*) is the mutation rate at time *t*. It is clear that *µ*(*t*) should depend on the mutation number within a cell. Denote the time point of the *i*-th mutation by *t_i _*(let *t*_0 _= 0). Thus during the small interval [*t_i_*−1, *t_i_*] the value of function *µ*(*t*) is

$$\mu \left(t\right)\approx \mu \left(t_{i}\right)\equiv \mu_{i}.$$

Based on the assumption of the Poisson process, we neglect the probability of two or more events taking place in (t, t+dt) as *dt *→ 0. If a cell is in state *p_i _*at time *t_i_*, the probability of transformation to state *p_i_*+1 in a small time interval Δ*t *is given by

$$\text{Prob}=\mu \left(t_{i+1}\right)\text{Δ}t+o\left(\text{Δ}t\right),$$

where *o*(Δ*t*)/Δ*t *→ 0 as Δt → 0. In addition, the probability of transformation from state *i *to state *i *+ *j *(with *j *> 1) in time Δ*t *is assumed to be *o*(Δ*t*). This implies that 1/*µ_i_*+1 is the averaged time required for a cell to go from state *i *to state *i *+ 1.

The probability to find a cell in the *i^th ^*stage by the end of time interval (*t, t *+ *dt*) is then given by

$$p_{i}\left(t+dt\right)=\left(1-\mu_{i+1}dt\right)p_{i}\left(t\right)+p_{i-1}\left(t\right)\mu_{i}dt$$

Taking the limit *dt *→ 0, the above equation becomes

(4) $$\frac{dp_{i}\left(t\right)}{dt}=\mu_{i}p_{i-1}\left(t\right)-\mu_{i+1}p_{i}\left(t\right),\;\;\;\;\;i=0,1,2,...$$

The initial condition *p_i_*(0) = 0 (i = 1, 2, · · · ) means that all the cells are normal at time *t *= 0.

In this section, we first present an explicit formula that includes the two formulae discussed in the previous section as examples. Following the notations introduced in the previous section, we also assume that the mutation rate *µ_i _*= *µ*(*t_i_*) is an increasing function of time *t*, where *t_i _*is the time when the *i^th^*-event occurs for *i *= 1, 2, · · ·, *N*.

For Eq. (4), we assume that the mutation rate *µ*(*t*) have the form of

$$\mu \left(t\right)=u_{0}e^{ast},$$

where *s *is the selective advantage coefficient and *u*_0 _the initial mutation rate. Here we propose to use a new coefficient *a *that is the transform factor linking the selective advantage coefficient and the mutation rate. Note that both *s *and *a *vary with individuals and the value of product *b *= *as *is determined by the curvature of *µ*(*t*). Thus we have

$$\mu_{j}\leq \mu_{j+1}.$$

Since *µ_j _*− *µ_j_*+1 = *µ*(*t_j_*) − *µ*(*t_j_*+1) usually is small, we have that

$$\begin{array}{cc} \mu_{j}p_{j-1}-\mu_{j+1}p_{j} & =\left(\mu_{j}-\mu_{j+1}\right)p_{j}+\mu_{j}\left(p_{j-1}-p_{j}\right)\\ & \approx \mu \left(t\right)\left(p_{j-1}-p_{j}\right) \end{array}$$

for *t_j_*−1 ≤ *t *≤ *t_j _*. Thus we have the following approximation

(5) $$\frac{dp_{j}}{dt}=\mu \left(t\right)\left(p_{j-1}-p_{j}\right),\;\;j=1,2,\cdots \;,N$$

for *t_j _*≤ *t *≤ *t_j_*+i. Let $\lambda \left(t\right)=\int_{0}^{t}\mu \left(x\right)dx$. Then we have the solution

$$p_{j}\left(t\right)=\frac{\lambda {\left(t\right)}^{j}e^{-\lambda \left(t\right)}}{j!}$$

for the system (5), where

$$\lambda \left(t\right)=\frac{\mu_{0}}{as}\left(e^{ast}-1\right).$$

And we further have

(6) $$t_{k}=\frac{\text{ln}\left(\frac{as\lambda_{k}}{\mu_{0}}+1\right)}{as}$$

for a new *k*-mutated cell $\lambda_{k}=\lambda (t_{k})$. That is, $p_{k}=\frac{1}{N}$, where *N *is the number of sensitive cells. Finally, we have that

(7) $$\lambda_{k}=-k.\;\text{LambertW}\left(-\frac{k{!}^{1/k}}{kN^{1/k}}\right)$$

where LambertW is the principal branch of the Lambert W function.

### The case of Beerenwinkel's formula

Beerenwinkel's formula mentioned above was based on their differential equations [26], [20] and [27]:

$$\frac{dp_{j}}{dt}=udp_{j-1}-\mu \left(t_{j+1}\right)p_{j}+sp_{j}\left(j-\left\langle j\right\rangle \right),$$

for *j *= 1, 2, · · ·, *N *, where $\left\langle j\right\rangle =\sum_{i}ip_{i}\left(t\right)$ is the average number of mutated loci at a given time *t*. This equation has a solution

$$p_{j}=\frac{\lambda^{j}e^{-\lambda }}{j!}$$

with $\lambda =\frac{ut\left(e^{st}-1\right)}{s}$[45], [46], [15], [47]. Since 〈*j*〉 = *λ*, we could write this equation as follows

$$\frac{dp_{j}}{dt}=ude^{st}\left(p_{j-1}-\mu \left(t_{j+1}\right)p_{j}\right),\;j=1,2,\cdots \;,N$$

Hence this equation becomes a special case of our equation (5) with *a *= 1 and *µ*_0 _= *ud*.

Note that the innovation od this work is the introduction of parameter *a*, which is important to make our model to be consistent with clinical data. If restricting *a *= 1, the predicted result was not supported by simulation [15]. In addition, Beerenwinkel's model assumed that each subsequent mutation has the same incremental effect on the fitness of the cell. It has been widely accepted that the impact of a specific mutation on phenotype will depend on the genetic background. For example, it was pointed out that the subsequent driver mutations appear faster and faster and the cumulative time to have *k *driver mutations grows with the logarithm of *k *[42]. Thus our equation depends on four parameters, namely the initial mutation rate *µ*_0_, selective advantage coefficient *s*, transforming factor *a*, and the number of driver genes. It is still in debate that which gene can be clarified as cancer candidate gene (CAN-gene). Sj*ö*blom et al defined 69 Can-genes [4], while Wood et al defined 142 CAN-genes [48]. If we look at 179854 colorectal cancer mutations in Cosmic v.64 statistics in [49], there were 340 patients who have more than 20 mutations. If we consider the top 100 highest mutation frequency genes as CAN-genes, the average number of the mutated CAN-gene is 29.23 per tumour. Since the average passenger mutation number in this study is around 200 per tumour, we choose the number of CAN-genes as *N *= 30 which covers the cases of more than 95% patients.

### Determination of waiting time *λ_k _*for cancer progression

Using Eq. (7), we calculated the values of model parameters for determining the values of waiting time *λ_k _*for three patients. The first patient Mx34 was 83 years old when she developed an advanced carcinoma of the ascending colon that was 9cm in diameter and of stage T4N2M1. A residual adenoma that surrounded the carcinoma was identified at the time of surgery. A laboratory detection showed that there were 17 mutations in colorectal adenoma and total mutations were 25 [1]. We assume that the average cell division time is 4 day (96 hours) and define a cell with 3 driver mutations to be adenoma and 12 mutations to be carcinoma. Similar to the data for patient Ma34 derived from [1], we produced the data in Table 1 which matched the estimation in [1] very well. We determined the model parameters in this case as *s *= 0.01, *a *= 0.32 and *µ*_0 _= 2 ∗ 10^−5 ^(see Table 3). Thus the function of mutation rate is defined by

**Table 1:** Values of *λ_k _*for three patients.

k	λ*_k_*1	λ*_k_*2	λ*_k_*3	k	λ*_k_*1	λ*_k_*2	λ*_k_*3
1	10^−10^	10^−10^	10^−9^	9	0.3211	0.3211	0.415
2	1.41 · 10^−5^	1.41 · 10^−5^	5 · 10^−5^	10	0.45287	0.45287	0.57
3	0.000843	0.000843	0.00181	11	0.60523	0.60523	0.746
4	0.006999	0.006999	0.0124	12	0.776297	0.776297	0.94
5	0.0260517	0.02605	0.04128	13	0.9642	0.9642	1.15
6	0.064499	0.0644	0.0946	14	1.16727	1.16727	
7	0.12599	0.12599	0.175	15	1.38388	1.38388	
8	0.211684	0.2116	0.282	16	1.61264	1.61264	

*λ_k_*_1_: patient Mx34 with *N *= 10^10^; *λ_k_*_2_: patient Mx32 with *N *= 10^10^; *λ_k_*_3_: patient Co82 with N = 10^9^.

**Table 2:** Tumour stage, waiting time (WT) (years) and number of mutations (NM) for patient Mx34 based on our calculation, and data published in [1].

Stages	WT	**WT in **[1]	NM	**NM in **[1]
Microadenema	1-2	3	3	3
Small-adenoma	3-4	5	4	4
Large adenoma	7	7	5	5
early carcinoma	8-19	8-23	6-25	6-25
Advanced carcinoma	20-30	25	> 25	> 25
Metastasis	33	25	> 33	28

**Table 3:** Selective advantage, initial mutation rate, waiting time for patients Mx34 and Co82.

Patient	*s*	*µ*_0_	*a*	*t_k_*
Mx34	0.01	2 ∗ 10^−5^	0.316	3165
Co82	0.0075	2 ∗ 10^−7^	0.309	3150

$$\mu \left(t\right)=\mu_{0}e^{ast}=2*10^{-5}\cdot e^{0.0032t},$$

which was not revealed in [1]. The determined time required to reach different tumour stages is listed in Table 2 for patient Mx34. For comparison, we also provide the prediction published in [1]. Table 2 suggests that our calculated results are consistent with the published ones.

This proposed time scheme describes how the advanced cancer evolved from the normal cells. However, we note that the first one or two driver mutations may not transform a normal cell into a cancer cell. As Jones et al described in [1], the APC/*β*-catenin passway mutations may only produce microadenoma. Even when all APC, Coca/Cin, KRAS, BRAF genes were mutated, only a large adenoma was produced. But it was still not yet necessary to be carcinoma.

Next we consider the case of patient 10 (patient Co82) in the dataset in [23]. The patient was 80 years old when she developed an advanced carcinoma of the cecum of stage T3N1M0. Evaluation of the same mutations in the large adenoma from which the carcinoma developed revealed the value of *F_LAd,ACa _*in (3) as 0.45. Application of Jones's Equation indicated that the large adenoma founder cell was born 36 years before the advanced carcinoma founder cell. Our proposed model suggests that the parameters in the mutation rate are *µ*_0 _= 10^−7^, *s *= 0.0075, and *a *= 0.309 (see Table 3). Thus the mutation rate is defined by

$$\mu \left(t\right)=10^{-7}e^{0.0016t}.$$

The time schedule is listed in Table 1. Note that the initial mutation rate of patient Mx34 (2^−5^) is close to the value of 3.4 · 10^−5 ^estimated by Bozic et al [42]. However, the initial mutation rate for patient Co82 is as little as 10^−7 ^which is the value estimated by Jackson et al in [50]. The determined time required to reach different tumour stages is listed in Table 4 for patient Co82. Comparing the results of these two patients, we see that patient Co82 was much healthier than patient Mx34, namely patient Co82 had a higher value of mutational robustness. To receive a good medical treatment outcome, different treatment schemes should be applied to these two patients.

**Table 4:** Tumour stage, time required (years) and number of driver mutations *k *for patient Co82 from our calculation.

Tumour stages	Time required	*k*
Microadenema	11	3
Small-adenoma	18	4
Large adenoma	22	5
carcinoma	27-33	6-11

### Estimation of relationship between tumour size and doubling time

Another application of our proposed formula is to establish the relationship between the number of driver mutations, the time spent to reach these driver mutations and the tumour size. We first consider patient Mx34 who was 83 years old when she developed an advanced carcinoma of the ascending colon that was 9 cm in diameter and of stage T4N2M1. A small mesenteric lymph node metastasis (1 cm in diameter) was found to contain 25 mutations that were subsequently evaluated in other lesions of this patient. Of these, 24 were found in the colorectal carcinoma (*F_ACa,Met _*= 0.04). The evaluation of the same mutations in the large adenoma from which the carcinoma developed revealed an *F_LAd,ACa _*of 0.23. Application of Eq. (3) indicated that the large adenoma founder cell was generated 17 years before the advanced carcinoma founder cell. In the 17 years between the birth of *F_cellLad _*and *F_cellACa_*, the tumour underwent waves of clonal expansion driven by mutations in TP53 and the other genes presumably required for invasion and further growth of this tumour. Once it acquired these capabilities, a cell (*F_cellMet_*) that is capable of lymph node metastasis appeared within a relatively short period. By using our model (5), we calculated that the averaged doubling time of tumour cells for patient Mx34 is 150 generations or about one year. This result is consistent with the prediction in Jones et al [1] that suggested that the mean doubling times are generally 2-4 months in metastases and much shorter in adenomas and carcinomas. For patient Co82, we also calculated the doubling time of tumour cells is 221 generations or about 1.5 years, which is also consistent with the predictions in [1]. All the calculation results are presented in Table 5.

**Table 5:** The number of driver mutations (*k*), time required (years) and number of cancer cells (NCCs).

*k*	time required	NCCs	*k*	time required	NCCs
8	13	6800	2	11	6.8
11	16.6	2 · 10^6^	3	18	300
15	19.4	10^8^	4	22	10000
19	21.3	10^9^	5	25	10^5^
26	23.5	5.5 · 10^9^	7	28	8 · 10^5^
30	24.4	8 · 10^9^	8	30	10^7^
33	25	9 · 10^9^	11	36	5 · 10^7^

1). The left three columns are for patient Mx34. 2), while the right three columns for patient Co82.

### Application to pancreas cancer

Yachida et al investigated seven pancreas cancer patients regarding the occurance of distant metastasis [43]. To distinguish between the possibilities that clonal evolution occurred inside the primary cancer and those within secondary sites, we sectioned the primary tumours from two patients into numerous, three-dimensionally organized pieces and examined the DNA from each piece for each of the founder and progressor mutations. For example, in patient Pa08, there were three progressor mutations present in two independent peritoneal metastases (defined as one subclone) and 23, 25, or 27 additional progressor mutations present in liver and lung metastases (defined as three additional subclones). Through the analysis of distinct regions of the primary tumour, it is clear that subclones giving rise to each of these metastases were present in the primary tumour. Moreover, these subclones were not small. From the size of the pieces and the amount of DNA recovered, each subclone should contain in excess of 100 million cells. In addition, more than four different subclones, each containing a similar large number of cells, could be identified through the analysis of other pieces of the same tumour. These subclones could be put into an ordered hierarchy establishing an evolutionary path of tumour progression. In addition, analysis of multiple primary tumour pieces and metastatic lesions from patient Pa04 revealed a similar clonal evolution, with distinct, large subclones within the primary tumours giving rise to the various metastases. By using the same raw data, our proposed model predicted that different patient should have different selective advantage coefficients and different initial mutation rates. The results are given in Table 6. Compared with the predicted selective advantage coefficient in [42], which is *s *= 0.004, our predicted value is slight larger than but still consistent with that prediction.

**Table 6:** Estimates of number of mutations (NMs), initial mutation rate (*µ*_0_) and selective advantage coefficient *s *for seven pancreas cancer patients

Patients	NMs	*µ*_0_	*s*
Pa01c	49	5 · 10^−5^	0.007
Pa02c	35	9 · 10^−5^	0.008
Pa03c	28	6 · 10^−4^	0.014
Pa04c	34	9 · 10^−5^	0.008
Pa05c	28	4 · 10^−4^	0.01
Pa07c	50	2.5 · 10^−4^	0.008
Pa08c	35	5 · 10^−5^	0.007
Average	37	2.2 · 10^−4^	0.0088

## Conclusions

Cancer progression essentially is a stochastic process. Statistical analysis is an important mathematical tool to analyze the progress of cancer cells based on a large number of cells in a particular position of the human body. In this work we proposed a new approach for analyzing the cancer progression in individuals. The developed model can be used to calculate the waiting time for carcinogenesis. Our model assumes that the expected waiting times depend on the values of three parameters, namely the selective advantage coefficient s, the transform factor a and the initial mutation rate *µ*_0_. Comparing with the Novak-Beerenwinkel model [15,20,26,27], we introduced the transform factor as a new parameter which makes our model more flexible. Thus our model is capable of matching with different mutational curves. In addition, our new model can be used to reveal the values of initial mutation rate, the selective advantage coefficient of tumour cells and the subsequent clonal expansion for individual patients. Our approach showed that, if the averaged mutational rate is known, a number of other biological parameters, such as the initial mutational rate and the mutational function, can be determined by using our proposed model. These parameters are important for constructing appropriate clinical treatment schemes for individual patients. The predicted values of parameters from our proposed model are consistent with the published ones in literature, which partially validated our model. The first example is the mean doubling time. We showed that the mean doubling time is one year for patient Mx34 and 1.5 years for patient Co82, which are consistent with the predictions in [1]. These results suggested that the mean doubling time in metastases, which is generally 2-4 months, should be much shorter than that in adenomas and carcinomas [1]. The second example is the selective advantage coefficient. Our model suggested that the selective advantage coefficient in colon cancer is about 0.01 ~ 0.0075 in Tables 3 and about 0.007 ~ 0.014 for pancreas caner in Table 6 which is close to but a little higher than the value of 0.004 that was estimated in [42].

Finding the values of initial mutation rate and the selective advantage coefficient also have potential applications in other related issues. For example, a mathematical approach has been designed to investigate the targeted cancer therapy recently [37,47,51,52]. The targeted cancer therapies use drugs that interfere with specific molecular structures implicated in tumour development [53]. The majority of the targeted therapies are either small-molecule drugs that act on targets inside the cell (usually protein tyrosine kinases) or monoclonal antibodies directed against tumour-specific proteins on the cell surface [54]. It has been showed that the overall probability *P *of tumour eradication as *P *= *P*_1_*P*_2_*P*_3_. Here *P*_1_, *P*_2 _and *P*_3 _are the probabilities that no resistance mutation leading to treatment failure arises during expansion, during steady state and during treatment, respectively [55].

The key parameters in this probability model are the number of tumour cells at steady state, time that the tumour remains at steady state before treatment, initial rate of cell division, initial death rate of tumour cells, the rate of resistance mutations, and the division and death rates (*r′ *and *d′*, respectively) of sensitive cells under treatment, in the absence of density constraints [55]. Note that (*d′ *− *r′*) is the selective advantage coefficient. This coefficient and the initial mutation rate can be calculated by using our formula. Hence our proposed model may have potential applications to design the targeted cancer therapy.

## Competing interests

The authors declare that they have no competing interests.

## Authors' contributions

SS and TT conceived and designed the study. SS and TT developed algorithms and carried out research. SS, FK and TT analyzed the data, interpreted the results and wrote the paper. All authors edited and approved the final version of the manuscript.

## Declarations

The publication costs for this article were funded by the corresponding author. This article has been published as part of BMC Systems Biology Volume 8 Supplement XXX, 2014: Selected articles from the IEEE International Conference on Bioinformatics and Biomedicine 2013: Systems Biology. The full contents of the supplement are available online at http://www.biomedcentral.com/bmcsystbiol/supplements/8/SXXX.

This article has been published as part of *BMC Systems Biology *Volume 8 Supplement 3, 2014: IEEE International Conference on Bioinformatics and Biomedicine (BIBM 2013): Systems Biology Approaches to Biomedicine. The full contents of the supplement are available online at http://www.biomedcentral.com/bmcsystbiol/supplements/8/S3.
